# Detection of Cortical Arousals in Sleep Using Multimodal Wearable Sensors and Machine Learning

**DOI:** 10.21203/rs.3.rs-6574148/v1

**Published:** 2025-05-16

**Authors:** Murat Kucukosmanoglu, Sarah Conklin, Kanika Bansal, Sena Kaya, Yumna Anwar, Quang Dang, Golshan Kargosha, Justin Brooks, Cody Feltch, Nilanjan Banerjee

**Affiliations:** D-Prime LLC; D-Prime LLC; University of Maryland; University of Maryland; University of Maryland; University of Maryland; D-Prime LLC; D-Prime LLC; Tanzen Medical Inc; University of Maryland

**Keywords:** cortical arousals, RestEaze, ADHD, wearables, machine learning, sleep monitoring

## Abstract

Cortical arousals are brief brain activations that disrupt sleep continuity and contribute to cardiovascular, cognitive, and behavioral impairments. Although polysomnography is the gold standard for arousal detection, its cost and complexity limit use in long-term or home-based monitoring. This study presents a noninvasive machine learning based framework for detecting cortical arousals using the RestEaze^™^ system, a leg-worn wearable that records multimodal physiological signals including accelerometry, gyroscope, photoplethysmography (PPG), and temperature. Across multiple methods tested, including logistic regression, XGBoost, and Random Forest classifiers, we found that features related to movement intensity were the most effective in identifying cortical arousals, while heart rate variability had a comparatively lower impact. The framework was evaluated in 14 children with attention-deficit/hyperactivity disorder (ADHD) who were being assessed for possible restless leg syndrome related sleep disruption. The Random Forest model achieved the best performance, with a ROC AUC of 0.94. For the arousal class specifically, it reached a precision of 0.57, recall of 0.78, and F1-score of 0.65. These findings support the feasibility of wearable-based machine learning for real-world arousal detection, demonstrated here in a pediatric ADHD cohort with sleep-related behavioral concerns.

## Introduction

Cortical arousals are brief interruptions in electroencephalographic (EEG) activity that fragment sleep without full awakening. Although transient, these arousals contribute to autonomic activation and disrupted sleep pattern, with growing evidence linking them to hypertension, cognitive decline, and elevated cardiovascular risk^1–3^. Total sleep duration less than 5 hours per night is considered high-risk for cardiovascular morbidity and mortality^4^. Disrupted or insufficient sleep has also been associated with systemic inflammation, metabolic dysfunction, and increased all-cause mortality^5^. Elevated rates of sleep disturbances, including cortical and autonomic arousals, have also been observed in children with attention-deficit/hyperactivity disorder (ADHD)^6–8^. Early and accurate detection of these arousals may offer clinical insights into the relationship between poor sleep quality and daytime behavioral symptoms that may reveal patterns that differ by clinical subtype.

Polysomnography remains the gold standard for detecting cortical arousals^9,10^, yet its high cost, complexity, and requirement for overnight clinical supervision limit its use for large-scale or long-term monitoring^11^. Consumer sleep technologies, such as sleep trackers, offer a non-invasive, scalable approach to sleep monitoring, with the potential to support early identification of sleep fragmentation in home environments. While these devices offer greater accessibility, they often suffer from poor agreement with polysomnography, particularly in detecting brief or motionless arousals^12^. A multicenter validation study involving 11 wearable, nearable, and airable consumer sleep trackers confirmed substantial variation in performance across devices, with some showing macro F1-scores as low as 0.26 when compared to Polysomnography^13^. However, the growing integration of wearable sleep technologies into daily life offers a valuable opportunity to develop advanced frameworks that can effectively use these technologies to detect clinically relevant features of sleep.

One promising solution involves tracking leg movements during sleep, which frequently occur alongside cortical arousals, especially in populations with conditions like restless leg syndrome, periodic limb movement disorder, or ADHD^14–17^. Recent studies using wearable leg sensors have shown that leg movements during sleep features can effectively distinguish arousals, and that leg-EEG signal coupling may reflect deeper physiological mechanisms of sleep disruption^18,19^. In this study, we evaluate multimodal sensor data from a leg-worn wearable, RestEaze^™^, to detect cortical arousals using interpretable machine learning models, with the aim of advancing practical and reliable sleep health monitoring solutions outside of traditional clinical settings.

The RestEaze^™^ system integrates accelerometry, gyroscope, photoplethysmography (PPG), and temperature sensors, offering a comprehensive view of movement and physiological dynamics during sleep. In a prior pilot study using a similar platform, we introduced neuro-extremity analysis, a novel approach that employed Granger causal modeling to assess the temporal and directional relationships between cortical arousals and leg movements^20^. That study revealed that textile-based capacitive sensors showed stronger temporal and spectral coupling with EEG-theta oscillations than inertial sensors, and more accurately identified expert-labeled cortical arousals. These findings support the hypothesis that leg movements and cortical arousals are driven by coordinated activity within a shared central arousal system. The current study builds upon this work by incorporating PPG and temperature sensors into the previously studied system and focusing exclusively on inertial sensors for movement detection, as they were found to reliably capture arousal-related leg movements while avoiding the redundancy and implementation challenges associated with textile-based capacitive sensors. This setup allows extraction of heart rate (HR) and heart rate variability (HRV) features that may offer additional insight into autonomic activation during sleep^21–23^.

## Results

Sleep is composed of two main states: rapid eye movement (REM) sleep and non-rapid eye movement (NREM) sleep. NREM includes three stages: N1, N2, and N3, which progress from light to deep sleep. These stages repeat in cycles throughout the night^24^. We began by examining the distribution of cortical arousals across sleep stages to establish a physiological context for the classification task. Arousals occurred most frequently during N2 sleep, with a mean proportion of 56.77% (95% confidence interval [CI]: 46.14–67.40%), followed by N1 at 17.47% (95% CI: 8.15–26.79%), REM at 13.17% (95% CI: 4.43–21.90%), and N3 at 12.60% (95% CI: 7.17–18.02%), averaged across subjects. This distribution aligns with established sleep physiology: N2 sleep not only comprises a larger portion of total sleep time but also has a lower arousal threshold, making it more prone to cortical arousals due to its transitional nature between wakefulness and deeper sleep stages^24^. Similarly, the elevated rate of arousals during N1 reflects its light sleep status and proximity to wakefulness. Interestingly, we also observed notable levels of arousals during N3 and REM sleep, suggesting increased cortical arousal beyond the lighter stages. This pattern may support prior findings showing that adolescents with ADHD and learning disorders exhibit increased cortical arousal during N2 and N3 sleep, particularly in central and frontal brain regions^25^.

To enable real-time detection of these arousal events using wearable data, we implemented and evaluated machine learning models designed to classify arousals from multimodal physiological signals. We evaluated the performance of three machine learning classifiers: Logistic Regression, XGBoost, and Random Forest for detecting cortical arousals based on multimodal physiological data from a leg-worn wearable device on full cohort of 14 children with ADHD, a population known to experience elevated levels of sleep fragmentation and frequent cortical arousals^6^. We chose these models to represent different levels of complexity and explainability: Logistic Regression as a simple linear baseline, Random Forest as a robust ensemble method, and XGBoost as a state-of-the-art gradient boosting algorithm.

All models were trained using a leave-one-subject-out cross-validation (LOOCV) approach to ensure robust subject-independent evaluation. The classification task involved identifying arousal events versus non-arousal periods. Evaluation metrics included class-wise precision, recall, F1-score, and overall Receiver Operating Characteristic - Area Under the Curve (ROC-AUC).

### Model training and performance

The performance of each model is summarized in Table 1. While all three classifiers showed high accuracy in detecting non-arousal periods (Class 0), their ability to detect arousal events (Class 1) varied considerably. Logistic Regression achieved a Class 1 F1-score of 0.57 and a ROC-AUC of 0.90. XGBoost improved precision but had lower recall for Class 1, with a resulting F1-score of 0.61 and a ROC-AUC of 0.93. Random Forest achieved the best balance, with a Class 1 F1-score of 0.65 and the highest ROC-AUC of 0.94. Based on these results, the Random Forest model was selected for further analysis. Table 1 summarizes the performance of each model.

## Feature Importance

**Figure 1:**
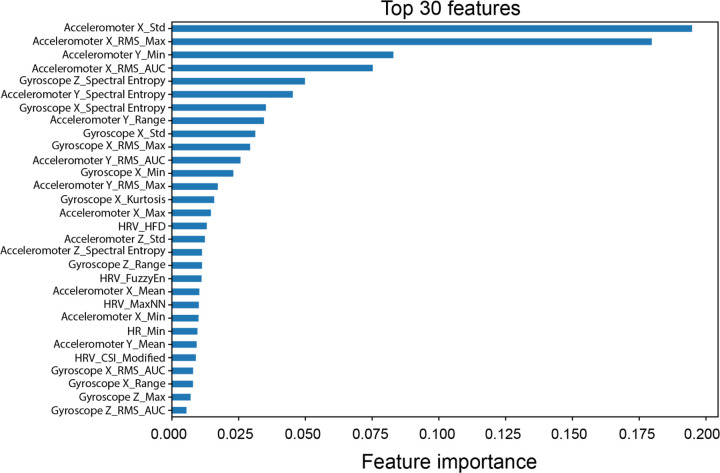
Top 30 Features for cortical arousal classification. Top features ranked by importance using a Random Forest model. Feature importance was determined based on the mean decrease in impurity.

Figure 1 presents the ranked list of the most important features contributing to cortical arousal classification, as determined by the Random Forest model. These features were predominantly derived from accelerometer and gyroscope signals, with a smaller contribution from HR and HRV metrics. The most important features included statistical, energy-based, and entropy-related measures. Importantly, standard deviation, root mean square (RMS), maximum, and range from the x-axis of the accelerometer appeared prominently in the ranking. This suggests that lateral leg movement (x-direction) plays a critical role in arousal episodes, consistent with biomechanical patterns observed during limb movement–related arousals.

Entropy-based features such as spectral entropy from both accelerometer and gyroscope signals were also among the top-ranked predictors. These features reflect the signal complexity or irregularity during sleep and are useful for capturing subtle variations in movement associated with arousals. Similarly, RMS AUC (Root Mean Square Area Under the Curve) quantifies cumulative signal energy, which is often elevated during microarousals due to brief bursts of leg activity.

Other contributing features included HRV-derived indices such as HRV Higuchi fractal dimension (HRV-HFD), HRV Cardiac Sympathetic Index (HRV-CSI), and HRV Fuzzy Entropy (HRV-FuzzyEn), all of which reflect beat-to-beat HRV complexity, physiological markers known to fluctuate during autonomic arousals^26^. However, they were less important than movement-based metrics, suggesting a stronger motor component to arousals in children with ADHD. Similarly, temperature-based features were not among the top-ranked predictors, indicating minimal relevance to arousal classification in this context.

In addition to feature rankings, we analyzed PPG signal quality across arousal categories. The mean PPG quality score was 0.818 (95% CI: 0.738–0.899) during non-arousal periods and 0.488 (95% CI: 0.420–0.556) during arousal events. This significant decline in signal quality during arousals suggests increased motion artifacts or sensor dropout, which may explain the lower importance of PPG-derived features in the final model.

### Agreement with Ground Truth

**Figure 2:**
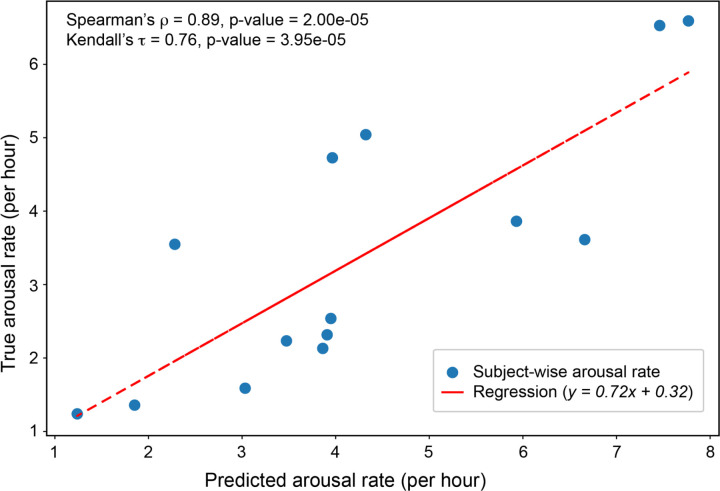
Arousal rate correlation. Correlation between predicted and true arousal rates (n = 14). Strong positive correlations were observed (Spearman’s ρ = 0.89, *p* = 2.00 × 10^−5^; Kendall’s τ = 0.76, *p* = 3.95 × 10^−5^). The solid line represents the best-fit linear regression: *y* = 0.72*x* + 0.32.

Figure 2 shows the model prediction of the arousal rates against the true arousal rates (ground truth). In this study, arousal rate refers to the number of 60-second windows that contain at least one cortical arousal event, normalized per hour of total sleep time. The predicted rates exhibited a strong correlation with the ground truth, yielding a Spearman’s rank correlation coefficient

$$\rho =0.89(\text{p}=2.00\times 10^{-5})\;\text{and}\;\text{a}\;\text{Kendall's}\;\tau =0.76(\text{p}=3.95\times 10^{-5}).$$

These results show a strong relationship, suggesting that the model successfully preserves subject-wise ranking in arousal frequency, which is crucial for estimating severity and comparing individuals.

The fitted linear regression line further supports the alignment between predicted and true values. The slope below 1.0 indicates underestimation at higher arousal rates, yet the close clustering of points around the line reflects consistency in the overall prediction trend. The regression slope was statistically significant (*p* < 0.01), with a 95% CI of [0.383, 1.050].

**Figure 3:**
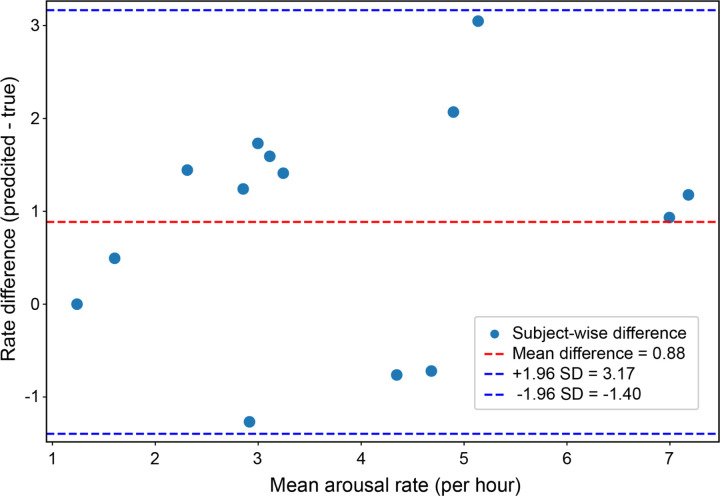
Bland–Altman plot for arousal rates. Bland–Altman plot comparing predicted and true (expert-labeled) arousal rates. The mean difference was +0.88 arousals per hour (Predicted − True), with 95% limits of agreement ranging from −1.40 to +3.17.

To further assess agreement, a Bland–Altman analysis was conducted (Fig. 3). This plot shows the differences between predicted and true arousal rates as a function of their average, both expressed in arousals per hour. The mean difference was + 0.88 arousals/hour (Predicted – True), indicating a slight overall tendency of the model to overestimate arousal frequency. The 95% limits of agreement ranged from − 1.40 to + 3.17 arousals/hour.

### Temporal Prediction Patterns

**Figure 4:**
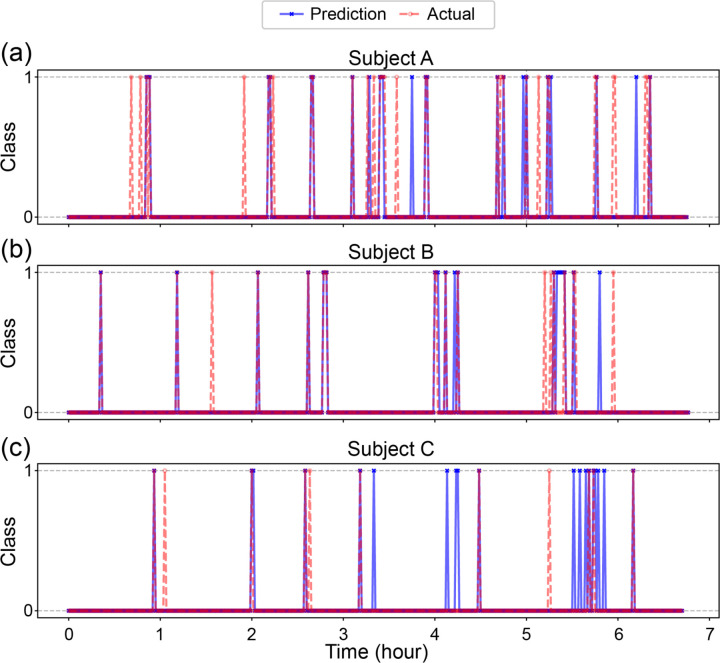
Temporal prediction of cortical arousals. Predicted versus true cortical arousal events for three ADHD participants. Each subplot shows 1-minute window predictions across the sleep period (x-axis in hours). Blue crosses represent model-predicted arousals, and red circles indicate ground truth events.

To evaluate model behavior across time, we visualized prediction sequences for three subjects who showed distinct arousal patterns. Figure 4 shows minute-by-minute comparisons between predicted and true arousals across the sleep duration.

For Subject A (Fig. 4a), who exhibited frequent and widely distributed arousals, the model effectively captured both isolated and clustered events throughout the night. Minute-by-minute inspection showed that most predictions were temporally aligned with ground truth, with several pre-arousal predictions appearing within one to two minutes of labeled events.

In contrast, Subject B (Fig. 4b) presented arousals that occurred in distinct temporal clusters during the early and late portions of the recording. The model maintained high temporal precision, correctly identifying contiguous arousal periods while avoiding false positives during quiescent intervals. Subject C (Fig. 4c) exhibited a sparser distribution of arousals. The model’s predictions closely matched the few true events, with overclassification toward the end.

The agreement between predicted and true arousals is quantified using Arousals (Class 1) F1-scores: 0.62 (a), 0.68 (b), and 0.54 (c). These scores indicate strong model performance given the substantial class imbalance, where arousals make up only ~ 6% of the data. For context, random guessing would yield an F1-score near 0.06, making the observed values highly meaningful. These subject-level, minute-by-minute visualizations highlight the model’s adaptability to inter-individual variability in sleep and arousal patterns.

## Discussion

This study demonstrates the feasibility of using multimodal wearable sensors and machine learning to detect cortical arousals during sleep, offering an accessible alternative to traditional in-clinic polysomnography. Among the classifiers tested, the Random Forest model achieved the best balance between recall and precision, yielding the highest ROC-AUC of 0.94. This result is consistent with Random Forest’s ability to handle complex patterns, feature interactions, and imbalanced data. Its ensemble-based architecture and embedded feature selection likely contributed to its robustness in this complex real-world dataset. Compared to Logistic Regression, which assumes linearity, and XGBoost, which can be sensitive to hyperparameter tuning in small datasets, the Random Forest model proved particularly effective at capturing subtle, subject-specific arousal signatures.

Feature importance analysis further revealed that the most predictive signals were derived from accelerometry and gyroscope data, particularly features reflecting signal variability and complexity, such as root mean square amplitude, standard deviation, and spectral entropy. These findings are consistent with prior work suggesting that leg movements are linked with cortical arousals^14,16,17^. Entropy measures likely captured the fragmented nature of movement during arousals. In contrast, HR and HRV features extracted from PPG contributed less prominently to model performance. This was not entirely unexpected, as the original sampling rate of 25 Hz may be insufficient for accurate HRV estimation. Prior work has shown that HRV metrics like Standard Deviation of NN Intervals (SDNN) and Root Mean Square of Successive Differences (RMSSD) require significantly higher sampling rates to ensure reliability, at least 50 Hz for SDNN and 100 Hz or more for RMSSD without interpolation^27^. Additionally, signal quality issues further limited the reliability of PPG-derived features. These noises, primarily motion artifacts and high-frequency noise, are inevitable in wearable-based health and well-being monitoring systems and can significantly impact peak detection accuracy^28^. In our study, the average PPG signal quality declined from 0.818 during non-arousal periods to 0.488 during arousal. This indicates a consistent drop in signal quality during arousal events.

Interestingly, the model predicted more arousals than were annotated by experts, particularly in subjects with sparse arousal profiles (Subject C). Rather than representing pure false positives, these predictions may reflect physiological events, such as sub-threshold arousals or autonomic activations, that were not captured by EEG-based criteria. This raises the possibility that wearable sensors may detect some physiological markers of sleep disruption that fall outside the boundaries of current clinical scoring systems. Indeed, prior research has shown that physiological changes surrounding arousal events can be significant, often extending beyond the boundaries of EEG-defined arousals^29,30^. These findings highlight how machine learning and wearables can improve sleep assessment beyond conventional methods. Additionally, the use of 60-second windows may have contributed to some discrepancy by grouping multiple arousals into a single event or capturing signal fluctuations surrounding true arousals.

Lastly, our subject-independent and interpretable framework provides minute-level temporal precision, making it suitable for clinical applications that require generalizable detection. It shows promise for individuals with ADHD, a group often underserved by traditional sleep diagnostics. Pediatric restless legs syndrome, for example, can cause significant sleep disruption, behavioral issues, and impaired daytime functioning that mimic ADHD symptoms^31,32^. While ADHD’s recognized subtypes (inattentive, hyperactive-impulsive, and combined) are well-described, their association with distinct sleep profiles remains unclear, highlighting the need for detailed pediatric sleep assessment^33^. Refined at-home monitoring could help identify specific sleep disorders and support more personalized, subtype-targeted treatments for pediatric ADHD. Building on these findings, this work presents multiple opportunities for future development. Priorities include expanding to larger and more diverse datasets, using deep learning to model long-range patterns, and incorporating continuous arousal scoring to reflect subtle physiological changes. Real-world feedback such as sleep staging, user experiences, and device usability will be vital for transforming this research into a practical home-based health solution. Ultimately, these efforts aim to bring clinical-quality sleep analytics into everyday environments through smart and accessible wearables.

## Conclusion

This study presents a non-invasive, wearable-based framework for detecting cortical arousals using multimodal physiological signals from a leg-worn device. Among the classifiers evaluated, the Random Forest model performed best, achieving a ROC-AUC of 0.94 and showing strong alignment with expert-labeled EEG annotations. Key predictive features, such as leg movement variability and signal entropy, support the role of movement-related physiological signals as markers of central arousals. These findings demonstrate the potential of systems like RestEaze^™^ for clinically meaningful, at-home sleep monitoring. Future work should include larger, more diverse populations and explore continuous arousal scoring to enhance clinical relevance.

## Methods

### Participants and Data Acquisition

**Figure 5:**
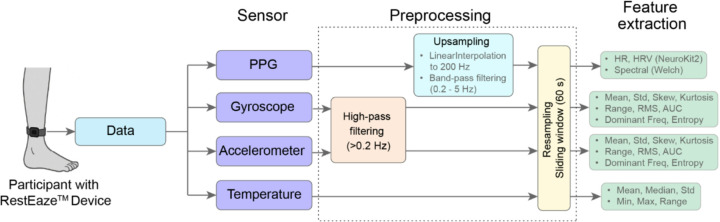
Multimodal data preprocessing pipeline for arousal classification. Raw data from the RestEaze^™^ wearable system included PPG, 3-D accelerometer, 3-D gyroscope, and temperature sensors

Physiological and movement data were collected from 14 children diagnosed with ADHD using the RestEaze^™^ Movement Analyzer, a wireless, leg-worn wearable designed for non-intrusive sleep monitoring and arousal detection. More details about the RestEaze^™^ can be found in previous publication^18^. As illustrated in Fig. 5, the RestEaze^™^ device integrates multiple synchronized sensors:

- A 3-D accelerometer and 3-D gyroscope embedded within an inertial measurement unit (IMU) for leg movement and orientation tracking,
- A PPG sensor for capturing cardiovascular dynamics, and
- Object and ambient temperature sensors for thermal signature during sleep.

The accelerometer (X, Y, Z axes), gyroscope (X, Y, Z axes), and PPG channels (IR, red, green LEDs) were all sampled at 25 Hz, providing high-resolution capture of biomechanical and cardiovascular signals. Temperature data was sampled at 0.2 Hz, appropriate for monitoring slow-changing thermal conditions.

This setup enables continuous, multimodal recording throughout the night, capturing both fine-grained leg movements and physiological fluctuations associated with cortical arousals. Across the 14 participants, the average total sleep time was approximately 7.59 hours per subject, totaling 106.32 hours of recorded sleep data. Data collection was conducted during natural sleep in a home or clinical setting.

All study procedures were approved by the Institutional Review Board of Johns Hopkins University. Research was conducted in accordance with the Declaration of Helsinki and all relevant ethical guidelines and regulations, including obtaining informed consent from all participants and/or their legal guardians.

### Cortical Arousals Rate

Cortical arousals (ground truth) were identified and scored according to the guidelines set by the American Academy of Sleep Medicine (AASM)^34^, which define arousals as abrupt shifts in EEG frequency, including alpha, theta, or activity exceeding 16 Hz, that last for at least 3 seconds and occur after a minimum of 10 seconds of uninterrupted sleep^21^. Arousal rate was calculated as the number of 60-second windows labeled with at least one cortical arousal event, normalized per hour of total sleep time. Specifically, if any arousal occurred within a given 60-second segment, the entire window was labeled as an arousal window (Class 1). The resulting arousal rate, expressed in arousal windows per hour, provides a temporally consistent metric for comparing arousal frequency across individuals.

In addition to cortical arousals, sleep stages, and limb movements were scored manually by trained technicians according to the AASM guidelines^34^. Bilateral limb movement events were also manually annotated, whereas leg movement channels were scored using an automated algorithm via the Sleepware G3 platform (Philips Respironics, US). Final scoring was reviewed and confirmed by a board-certified sleep physician and AASM fellow.

### Preprocessing and Feature Generation

All raw sensor signals were processed using a unified preprocessing pipeline (see Fig. 5), which included filtering, segmentation into 60-second non-overlapping windows, and modality-specific feature extraction. The choice of a 60-second window was guided by the need to balance temporal resolution with physiological interpretability. Each one-minute segment contains sufficient cardiac cycles (typically 60–100 beats) to allow reliable estimation of HR and HRV, while also being short enough to detect changes in physiological state over time.

For the PPG signal, the preprocessing began with upsampling to 200 Hz using linear interpolation. This step was essential for achieving the temporal resolution required for accurate peak detection and compatibility with feature extraction functions that assume higher sampling rates. Several methods did not perform at the native 25 Hz resolution, especially those involving frequency-domain HRV metrics. The upsampled signal was then bandpass filtered between 0.2 and 5 Hz using a Butterworth filter to remove baseline drift and suppress motion artifacts. The filter was implemented in Python 3.11 using the butter and filtfilt functions from the scipy.signal module, which apply zero-phase forward and reverse filtering to avoid phase distortion^35^.

**Figure 6:**
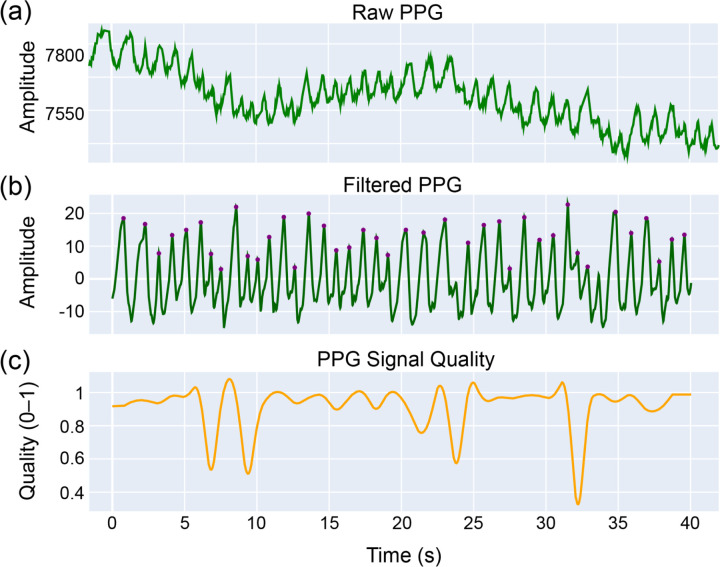
PPG signal preprocessing and peak detection. The top panel (a) shows the raw LED green PPG signal, which contains low-frequency drift and movement-related noise. The middle panel (b) displays the same signal after linear interpolation to 200 Hz and bandpass filtering (0.2–5 Hz). The bottom panel (c) shows the corresponding PPG signal quality over time, with values closer to 1 indicating cleaner, more reliable signal segments.

Following filtering, we evaluated several peak detection strategies to identify heartbeats from the PPG waveform. Among these, the ppg-findpeaks function from the NeuroKit2 library^36^ provided reliable results in terms of peak timing consistency and robustness to signal noise. Figure 6 shows the effects of preprocessing: the top panel displays the raw PPG signal with notable baseline fluctuations (Fig. 6a), the middle panel shows the filtered waveform with clearly resolved peaks (Fig. 6b), and the bottom panel plots the computed PPG signal quality over time (Fig. 6c). This quality metric, ranging from 0 to 1, reflects the reliability of the signal for physiological analysis.

Once peaks were detected, HR and HRV features were extracted from each 60-second window. HR metrics included minimum, maximum, and mean HR. HRV features encompassed time-domain measures (e.g., RMSSD, SDNN), frequency-domain indices (e.g., low-frequency/high-frequency ratio), and nonlinear metrics such as entropy, coefficient of signal irregularity, coefficient of variation of intervals, and fractal complexity (e.g., Higuchi fractal dimension).

Signals from the 3-D accelerometer and 3-D gyroscope were high-pass filtered with a cutoff frequency of 0.2 Hz to reduce low-frequency drift and artifacts. Each axis (X, Y, Z) was segmented into non-overlapping 60-second windows and processed to extract statistical features (mean, standard deviation, variance, skewness, kurtosis, minimum, maximum, and range), signal energy features (RMS and AUC), and spectral characteristics (dominant frequency and spectral entropy). Object and ambient temperature signals were not filtered but were similarly segmented into 60-second windows and processed to extract basic descriptive statistics, including mean, median, standard deviation, minimum, maximum, and range.

All features across modalities were combined into a unified feature matrix indexed by timestamp and subject ID. Arousal labels were resampled into 60-second non-overlapping windows to match the feature segmentation. A window was labeled as an arousal event if it contained any arousal occurrence within its duration, ensuring sensitivity to even brief arousal activity. This binary labeling approach allowed the model to learn from both isolated and clustered arousal events, supporting robust temporal prediction. The dataset was imbalanced, with arousal windows (Class 1) comprising 6.6% of the data and non-arousal windows (Class 0) accounting for 93.4%, reflecting the rarity of cortical arousals during sleep.

While this approach simplifies the classification task, it introduces a limitation: multiple arousals occurring within the same 60-second window are treated as a single event. This may underestimate the actual number of arousals in windows with dense activity. We initially experimented with shorter windows (e.g., 30 seconds) to capture finer temporal dynamics. However, this led to increased false positives, likely because pre- and post-arousal changes over the signals extended beyond the arousal itself. Thus, the 60-second window length was selected as an optimal trade-off between capturing relevant signal changes and maintaining specificity. Additionally, arousals that spanned multiple windows, a potential source of edge effects, were observed in approximately 10% of cases. Given that most arousals lasted 8 to 12 seconds, this level of boundary overlap was considered acceptable within the 60-second segmentation framework.

### Machine Learning Framework and Feature Selection

We evaluated and compared the performance of three classifiers:

### Logistic Regression

As a baseline, we trained a Logistic Regression model with L2 regularization (Ridge penalty), which helps prevent overfitting and handles multicollinearity. The model was trained with subject-level z-scored features, class balancing, and LOOCV. Hyperparameters, including the regularization strength, were tuned using RandomizedSearchCV with 50 randomized iterations. While it offers greater interpretability, it lacks the capacity to model nonlinear interactions present in physiological time-series data.

### Gradient-Boosted Decision Tree Model (XGBoost)

We also implemented XGBoost, a high-performance gradient-boosted decision tree model that incorporates both first- and second-order gradients. We tuned hyperparameters including learning rate, tree depth, subsampling rate, and L1/L2 penalties using RandomizedSearchCV with 50 randomized iterations. All training followed the same LOOCV protocol as the previous model.

### Bagged Tree Ensemble Model (Random Forest)

We used a Random Forest classifier, known for its robustness to noise, ability to model nonlinear relationships and embedded feature importance analysis. Hyperparameters were optimized using RandomizedSearchCV with 50 randomized iterations. Tuned parameters included the number of trees, maximum depth, minimum samples per split and leaf node, and feature subsampling ratio. All training followed the same LOOCV protocol as the other models. The best-performing hyperparameters for each model, selected based on cross-validation performance across folds, are summarized in Table 2.

To account for inter-individual variability in physiological signals, all features were standardized per subject using z-score normalization. Columns with excessive missingness were removed, and the remaining missing values were imputed using subject-level k-nearest neighbors^37^. This method estimates missing values by averaging the feature values from the most similar observations in the dataset. Dimensionality reduction and feature selection were performed using Recursive Feature Elimination^38^ within the training folds to retain only the most informative features for classification.

A LOOCV scheme was used, where each subject was held out in turn as the test fold while the remaining subjects were used for training. This approach ensured strict subject-level separation and prevented data leakage, supporting robust evaluation of model generalizability.

To address the natural class imbalance between arousal and non-arousal events, a two-step resampling strategy was applied within each training fold. First, Tomek Links^39^ were removed to clean the decision boundary, followed by Random Undersampling^40^ to balance the class distribution during model fitting. Importantly, the held-out test subject was never undersampled, preserving the original data distribution for evaluation. Thresholds for classification were selected based on the precision-recall curve computed on the raw (non-resampled) version of the training data, ensuring that decision thresholds reflected realistic class ratios. The selected threshold was then applied to the test fold.

Together, these classifiers enabled direct performance comparisons. The outputs were evaluated using window-based overlap metrics and correlation analyses, described in the next section.

### Model Comparison and Evaluation

Model performance was assessed using both classification-based metrics and agreement-based statistical analyses, with careful consideration given to subject-level separation through LOOCV. For each model, the area under the ROC-AUC was computed to quantify overall discriminative ability. In addition, precision, recall, and F1-score, defined in equations (1) through (3), were calculated separately for arousal (Class 1) and non-arousal (Class 0) classes on a per-window basis. These equations quantify the performance of the model in different aspects:

(1) $$\text{Precision}=\frac{\text{True}\;\text{Positives}}{\text{True}\;\text{Positives}+\text{False}\;\text{Positives}}$$

(2) $$\text{Recall}=\frac{\text{True}\;\text{Positives}}{\text{True}\;\text{Positives}+\text{False}\;\text{Positives}}$$

(3) $$F_{1}=2\frac{\left(\text{Precition}\;x\;\text{Recall}\right)}{\left(\text{Precition}\;+\;\text{Recall}\right)}$$

To ensure equal contribution from each subject and prevent performance estimates from being skewed by subjects with longer recordings or more events, all metrics (precision, recall, F1-score) were first computed individually for each left-out subject in the LOOCV framework. The final reported values (Table 1) represent the mean of per-subject metrics, formalized as:

(4) $$\bar{M}=\frac{1}{S}\sum_{s=1}^{S}M^{\left(s\right)}$$

Where:

$\bar{M}$ Subject-averaged metric (e.g., precision, recall, F1-score)

*S*

### Total number of subjects


$$M^{\left(s\right)}:\text{Metric}\;\text{value}\;(e.g.,\;\text{Precitio}n^{\left(s\right)}=\frac{\text{True}\;\text{Positive}s^{\left(s\right)}}{\text{True}\;\text{Positive}s^{\left(s\right)}+\text{False}\;\text{Positive}s^{\left(s\right)}}$$

In addition to discrete classification metrics, we evaluated the agreement between predicted arousals and ground truth arousals across subjects. The predicted arousal rate for each subject, defined as the number of arousal events per hour of total sleep time, was compared with the true arousal rate using Spearman’s rank correlation coefficient (ρ) and Kendall’s tau (τ) to assess monotonic relationships. Agreement between predicted and true arousal rate were further examined using Bland–Altman analysis^41^, which visualizes the bias and limits of agreement between model estimates and expert-scored references.

### Feature Importance Analysis

After model training and evaluation, we analyzed feature importances using the Random Forest model trained on the entire dataset to capture generalizable patterns across all subjects. Random Forest determines feature importance by evaluating the total decrease in node impurity, such as Gini impurity, each feature contributes across all decision trees in the ensemble. Features that result in larger impurity reductions when used for splitting are considered more important^42^. This approach allows the model to naturally account for nonlinear relationships and feature interactions. To enhance interpretability and reduce noise from low-importance variables, we selected the top ranked features for post hoc analysis. This number was chosen empirically: including more than 30 features resulted in only marginal improvements in classification performance while increasing model complexity and risk of overfitting. The selected features represented a balanced trade-off between performance and interpretability and were used in downstream visualizations and interpretation.

## Figures and Tables

**Figure 7:**
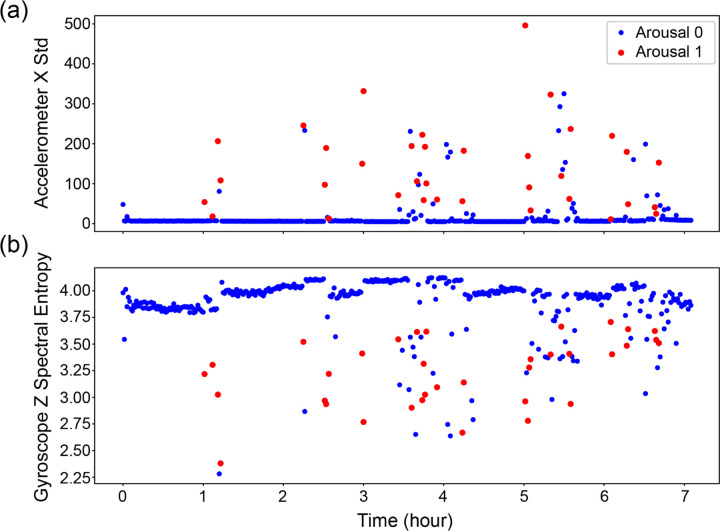
Accelerometer and gyroscope feature trends across sleep. The top panel (a) shows the standard deviation of the X-axis accelerometer signal, reflecting variability in leg movement amplitude. The bottom panel (b) displays the spectral entropy of the Z-axis gyroscope signal, which quantifies the irregularity or complexity of rotational motion. Red markers indicate windows labeled as arousals, while blue markers denote non-arousal periods.

**Table 1:** Model Performance Summary

Model	Class	Precision	Recall	F1-Score	ROC-AUC
Logistic Regression	0	0.99	0.94	0.96	0.90
	1	0.45	0.84	0.57	
XGBoost	0	0.99	0.95	0.97	0.93
	1	0.50	0.82	0.61	
Random Forest	0	0.99	0.96	0.98	0.94
	1	0.57	0.78	0.65	

**Table 2:** Best-performing Hyperparameters for Each Classifier

Classifier	Best Hyperparameters
Logistic Regression	C = 0.1336, penalty = ‘l2’, solver = ‘liblinear’, fit_intercept = True, max_iter = 1000, tol = 0.0039
XGBoost	colsample_bytree = 0.7547, gamma = 4.6836, learning_rate = 0.0513, max_depth = 6, n_estimators = 91, reg_alpha = 0.2579, reg_lambda = 2.9800, scale_pos_weight = 2, subsample = 0.9929
Random Forest	ccp_alpha = 0.0036, criterion = ‘entropy’, max_depth = 15, max_features = 0.3986, min_samples_leaf = 3, min_samples_split = 6, min_weight_fraction_leaf = 0.0037, n_estimators = 120

## Data Availability

All data generated or analyzed during the current study are not publicly available due to institutional restrictions but are available from the senior author (NB) upon reasonable request and with approval from the University of Maryland, Baltimore County.

## Abbreviations

AASM: American Academy of Sleep Medicine
ADHD: Attention-Deficit/Hyperactivity Disorder
AUC: Area Under the Curve
CSI: Cardiac Sympathetic Index (HRV-derived)
EEG: Electroencephalographic
FuzzyEn: Fuzzy Entropy (HRV-derived)
HRV: Heart Rate Variability
HFD: Higuchi fractal dimension (HRV-derived)
LOOCV: Leave-One-Subject-Out Cross-Validation
PPG: Photoplethysmography
REM: Rapid Eye Movement
NREM: Non-Rapid Eye Movement
RMS: Root Mean Square
RMS AUC: Root Mean Square Area Under the Curve
RMSSD: Root Mean Square of Successive Differences
ROC: Receiver Operating Characteristic
SDNN: Standard Deviation of NN Intervals
